# Progression of SARS-CoV-2 Seroprevalence in St. Louis, Missouri, through January 2021

**DOI:** 10.1128/mSphere.00450-21

**Published:** 2021-08-04

**Authors:** Brittany K. Smith, Andrew B. Janowski, Arim C. Fremont, Lucas J. Adams, Ya-Nan Dai, Christopher W. Farnsworth, Ann M. Gronowski, Stephen M. Roper, David Wang, Daved H. Fremont

**Affiliations:** a Department of Pathology & Immunology, Washington University School of Medicine, St. Louis, Missouri, USA; b Department of Pediatrics, Washington University School of Medicine, St. Louis, Missouri, USA; c Department of Biochemistry & Molecular Biophysics, Washington University School of Medicine, St. Louis, Missouri, USA; d Department of Molecular Microbiology, Washington University School of Medicine, St. Louis, Missouri, USA; University of Michigan—Ann Arbor

**Keywords:** COVID-19, SARS-CoV-2, seroprevalence, serology, ELISA

## Abstract

Severe acute respiratory syndrome coronavirus 2 (SARS-CoV-2) seropositivity was assessed for 3,066 individuals visiting hospitals in St. Louis, Missouri, during July 2020, November 2020, or January 2021. Seropositivity in children increased from 5.22% in July to 21.16% in January. In the same time frame, seropositivity among adults increased from 4.52% to 19.03%, prior to initiation of mass vaccination.

**IMPORTANCE** This study determined the percentage of children and adult samples from the St. Louis metropolitan area in Missouri with SARS-CoV-2 antibodies during three collection periods spanning July 2020 to January 2021. By January 2021, 20.68% of the tested individuals had antibodies. These results show the evolution of the SARS-CoV-2 pandemic in St. Louis, Missouri, and provide a snapshot of the extent of infection just prior to the start of mass vaccination.

## INTRODUCTION

Accurate estimates of severe acute respiratory syndrome coronavirus 2 (SARS-CoV-2) infection rates are vital in understanding viral spread and death rates and in planning vaccine distribution. Reported coronavirus disease 2019 (COVID-19) cases likely underestimate true prevalence due to a large number of mild and asymptomatic cases. Seroprevalence studies test for the presence of antibodies rather than virus (1) and can therefore inform cumulative infection rates in specified populations.

We previously reported an estimated seropositivity rate of 3.11% (95% credible interval [95% CrI], 0.92% to 5.32%) for adult patients presenting to Barnes-Jewish Hospital in St. Louis, Missouri, in April-May 2020 (2). The pediatric seropositivity rate was 1.71% (95% CrI, 0.04% to 3.38%) for St. Louis Children’s Hospital patients during a similar time frame (2). Here, we report estimated seropositivity rates for adult and pediatric serum/plasma samples collected during three additional collection periods between July 2020 and January 2021. Collectively, rates from the four time periods provide critical information on degree of community spread and the evolution of the pandemic in the St. Louis metropolitan area in Missouri.

## RESULTS

Collectively, between the three time frames, a total of 3,066 samples were tested, each from a unique patient. Of these samples, 1,552 were from pediatric patients from 2 days to 17 years old, with a median age of 10 years (Table 1). The adult cohort contributed 1,514 of the samples; the adults were 18 to 103 years old, with a median age of 61 years. Of the samples, 57.8% came from female patients, and 42.2% were from male patients. The age distribution of pediatric samples was similar to that of pediatric individuals in the St. Louis metropolitan area in Missouri. Among adult samples, individuals aged 65+ are overrepresented, making up 21.9% of the 18+ population but 39.7% of our adult samples, while individuals aged 18 to 39 years are underrepresented, consisting of 36.4% of the St. Louis adult population but 15.8% of our adult cohorts (2019 census).

**TABLE 1:** Demographic characteristics of pediatric and adult cohorts over three collection periods

Cohort and patient characteristic	July 2020		November 2020		January 2021	
	Total no. of patients	No. of seropositive patients (%)	Total no. of patients	No. of seropositive patients (%)	Total no. of patients	No. of seropositive patients (%)
Pediatric cohort						
Age (yr)						
<5	142	5 (3.52)	144	13 (9.03)	133	24 (18.05)
5−9	125	7 (5.60)	78	15 (19.23)	115	19 (16.52)
10−17	279	22 (7.89)	282	54 (19.15)	254	66 (25.98)
Sex						
Male	254	13 (5.12)	275	52 (18.91)	271	53 (19.56)
Female	292	21 (7.19)	229	30 (13.10)	231	56 (24.24)
Total	546	34 (6.23)	504	82 (16.27)	502	109 (21.71)
Adult cohort						
Age (yr)						
18−39	73	4 (5.48)	89	9 (10.11)	75	18 (24.00)
40−64	225	9 (4.00)	232	18 (7.76)	219	42 (19.18)
65+	208	15 (7.21)	188	9 (4.79)	205	38 (18.54)
Sex						
Male	161	5 (3.11)	196	16 (8.16)	190	50 (26.32)
Female	345	23 (6.67)	313	20 (6.39)	309	48 (15.53)
Total	506	28 (5.53)	509	36 (7.07)	499	98 (19.64)

**FIG 1:**
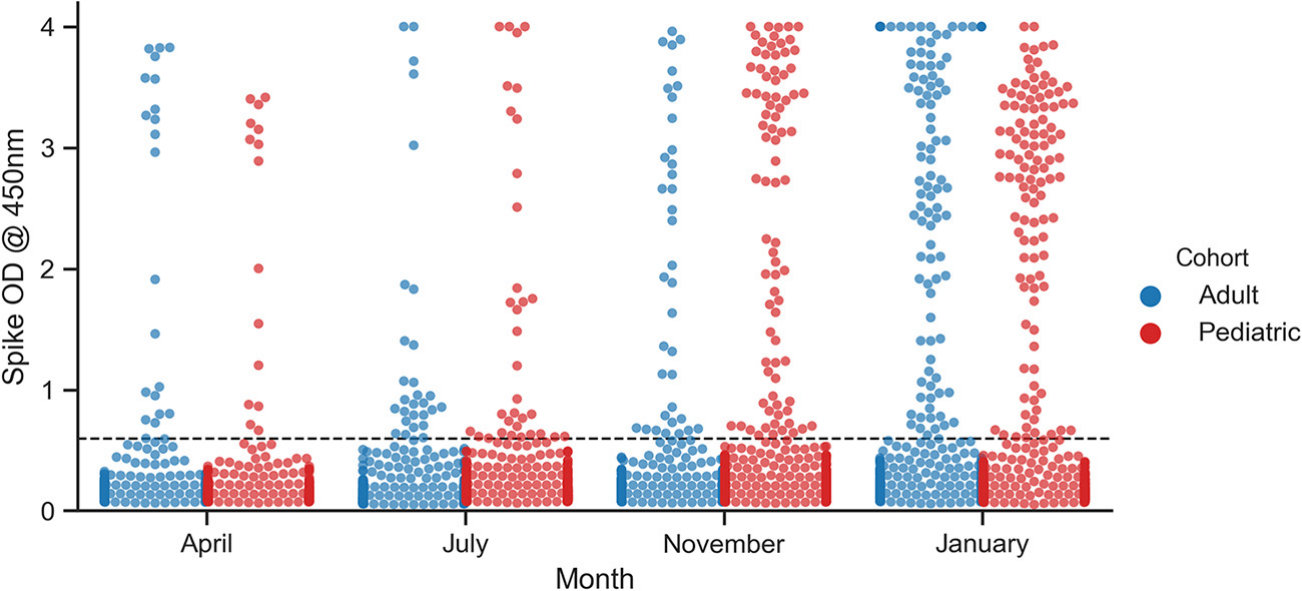
Distribution of spike IgG ELISA results for adult and pediatric cohorts during each collection window. The horizontal dashed line represents the cutoff for positivity. The cutoff was previously determined based on ROC analysis of 300 pre-COVID-19 and 110 RT-PCR-positive (RT-PCR+) serum samples (2).

Enzyme-linked immunosorbent assay (ELISA) results were aggregated with previously published April 2020 data (Fig. 1) (2). During April 2020, 36/1,055 (3.41%) samples were found positive for anti-spike IgG antibodies (Abs) with optical density (OD) values above the cutoff value (2). Throughout the subsequent collection periods, the fraction of positive samples across all age groups increased to 62/1,052 (5.89%), 118/1,013 (11.65%), and finally 207/1,001 (20.68%) in the final collection period during January 2021 (Table 1).

**FIG 2:**
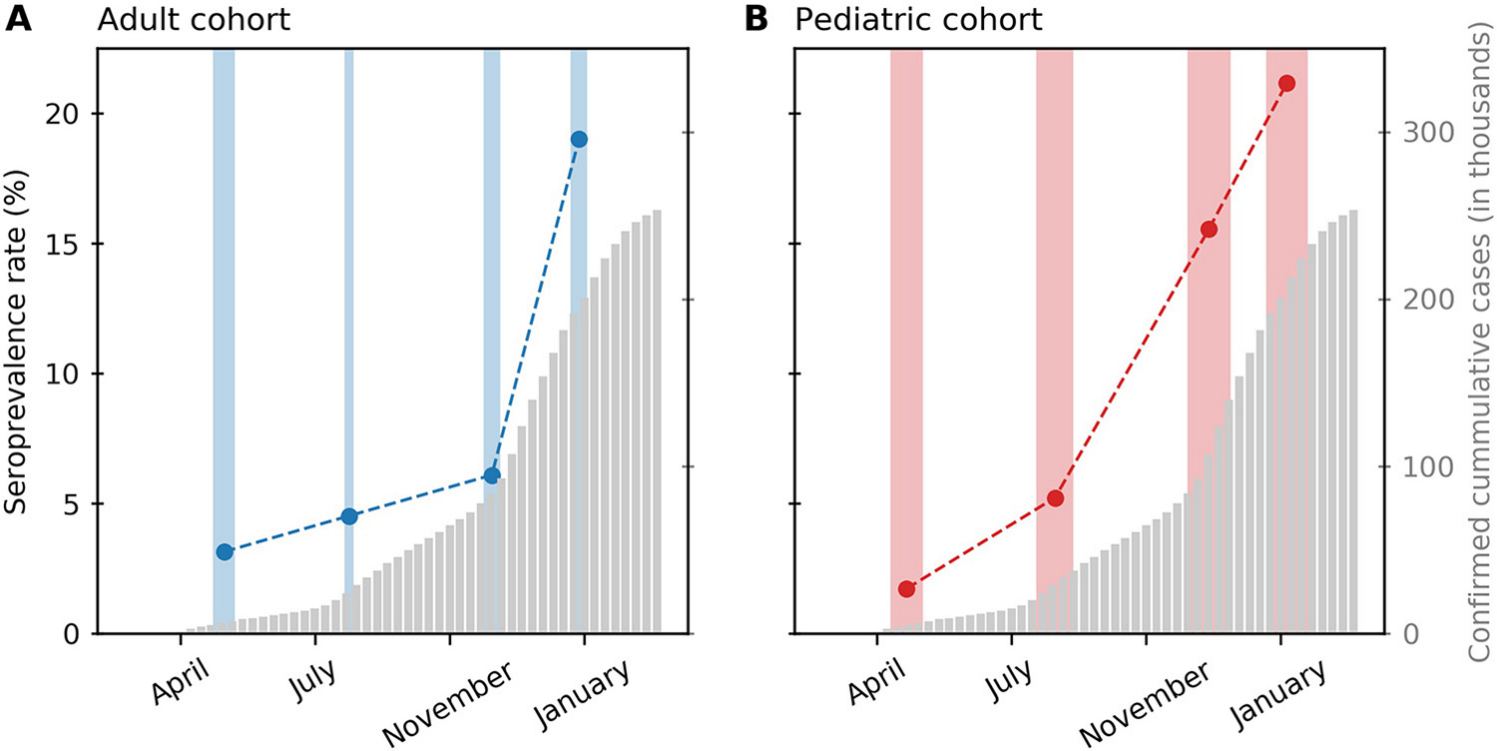
Seroprevalence estimates during each collection window for adult (A) and pediatric (B) cohorts. The cumulative number of laboratory-confirmed COVID-19 cases in the St. Louis metropolitan area is presented using gray bars (https://slu-opengis.github.io/covid_daily_viz/index.html).

Within the adult cohort, the estimated seroprevalence rate of our samples increased from 3.11% (95% CrI, 0.92% to 5.32%) in April 2020 to 4.52% (95% CrI, 2.07% to 6.93%), 6.09% (95% CrI, 3.45% to 8.73%), and then 19.03% (95% CrI, 15.24% to 22.80%) in July 2020, November 2020, and January 2021, respectively. Within the pediatric cohort, the estimated rate in April 2020 was 1.72% (95% CrI, 0.01% to 3.42%). The rate increased to 5.22% (95% CrI, 2.76% to 7.68%), 15.56% (95% CrI, 12.07% to 19.05%), and finally 21.16% (95% CrI, 17.21% to 25.09%) in July, November, and January (Fig. 2).

Our results do not provide evidence for seroprevalence rate differences based on sex during the first three collection periods but show significantly higher rates in males than females during January 2021 (adjusted rates, 24.74% versus 16.71%; *P* = 0.0057). Additionally, during the April 2020 collection period, we previously found a significantly lower positivity in young children (less than 5 years) than in adults (2). However, in each of the three collection periods assessed in this study, the positivity rates of children under 5 years old are similar to that seen in adults (*P* > 0.204).

Vaccination in St. Louis, Missouri, began on 14 December 2020. During the final adult collection window, ranging from 25 December 2020 through 5 January 2021, 0.96% to 2.05% of the St. Louis population had received at least one vaccine and 0% to 0.11% had received both doses (https://covidvaccine.mo.gov/data/); thus, vaccination is unlikely to have substantially impacted the seropositivity rate. To experimentally assess whether the adult January 2021 seroprevalence rate we report was affected by vaccines, we also tested these samples for reactivity to nucleoprotein (NP) (see Fig. S1 in the supplemental material) using an ELISA developed previously (2). Not all spike-positive samples were also positive by the NP assay; however, the fraction of spike-positive samples that were negative for NP was similar to the fraction observed for April 2020 samples, before vaccinations had been developed (*P* = 0.78).

10.1128/mSphere.00450-21.1 FIG S1 NP and spike IgG ELISA results for adult samples collected between 25 December 2020 through 5 January 2021. The horizontal and vertical dashed lines represent cutoff values for NP and spike assays, respectively. Download FIG S1, TIF file, 2.8 MB. Copyright © 2021 Smith et al. 2021 Smith et al. https://creativecommons.org/licenses/by/4.0/ This content is distributed under the terms of the Creative Commons Attribution 4.0 International license.

We also used NP assay sensitivity and specificity along with NP ELISA results in a Bayesian regression model to estimate the seroprevalence rate for January 2021 adult samples. The estimated rate based on the NP assay was 21.1% (95% CrI, 15.36% to 26.86%), largely overlapping with the seroprevalence rate we estimated using the spike assay, 19.03% (95% CrI, 15.24% to 22.80%). Therefore, while some vaccinated individuals may be included in our cohort, the low vaccination rate during the collection window and NP-based seroprevalence rate suggest that any potential effect of vaccination would be minimal.

## DISCUSSION

Our study estimates the prevalence of SARS-CoV-2 IgG antibodies in residual serum/plasma samples collected during three collection windows between July 2020 and January 2021, prior to mass vaccination in St. Louis, Missouri. We report seroprevalence rates for adult and pediatric cohorts during July 2020, November 2020, and January 2021 collection windows, aggregated with previously reported rates for April 2020.

Our study has several limitations. Samples are collected from patients obtaining medical care so are not necessarily generalizable to the St. Louis population. Additionally, collection dates for pediatric and adult patients are similar for each window, though not identical. Discrepancies between collection dates and inclusion criteria between pediatric and adult cohorts may limit comparative interpretation of the observed rates. Further, the kinetics of antibody waning over time is currently unclear (3, 4). Some studies have suggested waning antibody responses within only months following infection (3, 5), leading to potential underestimation in seroprevalence rates among samples collected in summer 2020 and later.

For samples collected in January 2021, the estimated seroprevalence rates are 19.03% (95% CrI, 15.24% to 22.80%) for adults and 21.16% (95% CrI, 17.21% to 25.09%) for the pediatric cohort. These rates are similar to the 18.9% seroprevalence rate found in patients receiving dialysis across 43 states in January 2021 (6).

Among pediatric samples, the greatest increase in prevalence between collection periods occurred between July and November (Fig. 2). However, for adult samples, there were modest changes in prevalence between April, July, and November collection periods, followed by a significant increase between November 2020 and January 2021. St. Louis experienced a peak in reported COVID-19 cases throughout the month of November, highest on 16 November 2020 (https://slu-opengis.github.io/covid_daily_viz/index.html). Because the midpoints of adult and pediatric November collection periods occurred on November 2 and November 15, respectively, the pediatric November data may have captured more of the peak in reported cases in St. Louis than the adult data.

We observe a seroprevalence rate of 20.02% across all January 2021 samples, over twice as high as the rate of reported cases (https://slu-opengis.github.io/covid_daily_viz/index.html). However, this seroprevalence rate remains substantially lower than that required to achieve herd immunity (7). Additional seroprevalence surveys are necessary to monitor the pandemic, including seroprevalence rates following mass vaccination.

## MATERIALS AND METHODS

**Patient cohorts.** We estimated the prevalence of SARS-CoV-2 antibodies in patients presenting to hospitals in the St. Louis metropolitan area in Missouri during three collection windows, spaced between July 2020 and January 2021. Adult samples were residual samples sent to the Barnes-Jewish Hospital for physician-ordered vitamin D testing. Over 90% of the samples were from outpatients, of which 66% were ordered from primary care physicians, 21% from patients at cancer centers, and 12% from patients prior to elective surgeries. The collection windows for adult samples were 25 July 2020 to 31 July 2020, 27 October 2020 to 7 November 2020, and 25 December 2020 to 5 January 2021, 499 to 509 samples were collected during each period. Pediatric samples included residual serum or plasma specimens from unique outpatients presenting to St. Louis Children’s Hospital who had blood drawn for screening for a variety of conditions (including orders such as bilirubin, vitamin D, iron/ferritin, thyroid-stimulating hormone [TSH]/free T4, and hemoglobin A1c [HgbA1c]). Collection windows for pediatric specimens were 22 July 2020 to 16 August 2020, 1 November 2020 to 30 November 2020, and 24 December 2020 to 21 January 2021, and 502 to 546 samples were collected during each period. This study was approved by the Human Research Protection Office at Washington University in St. Louis (202004199 and 202004153).

**SARS-CoV-2 spike and NP ELISAs.** Seropositivity for 3,066 samples was assessed using an in-house IgG ELISA based on spike antigen. Vaccination of adults in St. Louis, Missouri, began just prior to the January 2021 collection window so spike-positive samples were also tested for reactivity to nucleoprotein (NP). The development and validation of in-house IgG ELISAs based on NP and trimeric spike proteins were described previously (2). Optimal cutoffs were determined by receiver operating curve (ROC) analysis, using 300 serum samples collected in 2007 to 2008 as negative controls and 110 samples from COVID-19 reverse transcription-PCR (RT-PCR)-positive individuals as positive controls (2). Cutoff values were set by maximizing the Youden index. The cutoff for the spike ELISA was set as the average pre-COVID-19 OD value plus 4 standard deviations (SD), resulting in 98.2% sensitivity (95% CrI, 97.4% to 99.9%) and 98.7% specificity (95% CrI, 95.7% to 100%) The NP ELISA cutoff was set as the average pre-COVID-19 OD plus 2 SD, with 86.5% (95% CrI, 80.1% to 92.8%) sensitivity and 93.1% (95% CrI, 90.3% to 95.8%) specificity (2).

**Statistical analysis.** Seroprevalence rates were then estimated in a Bayesian regression model by accounting for sensitivity and specificity of the IgG ELISA. A Bayesian framework using a Markov chain Monte Carlo (MCMC) method was implemented in python using the PyStan package (8). Bayesian hypothesis testing was used to examine the effects of age and sex on SARS-CoV-2 seroprevalence. We report Bayesian *P* values (9), reflecting the χ^2^ discrepancy measure between generated data sets and expected values drawn from the posterior distribution.
