# Supplementary figures and images for: Progression of SARS-CoV-2 Seroprevalence in St. Louis, Missouri, through January 2021
Source: mSphere. 2021 Aug 4;6(4):e00450-21. doi: 10.1128/mSphere.00450-21 (PMC8386375; doi:10.1128/mSphere.00450-21)

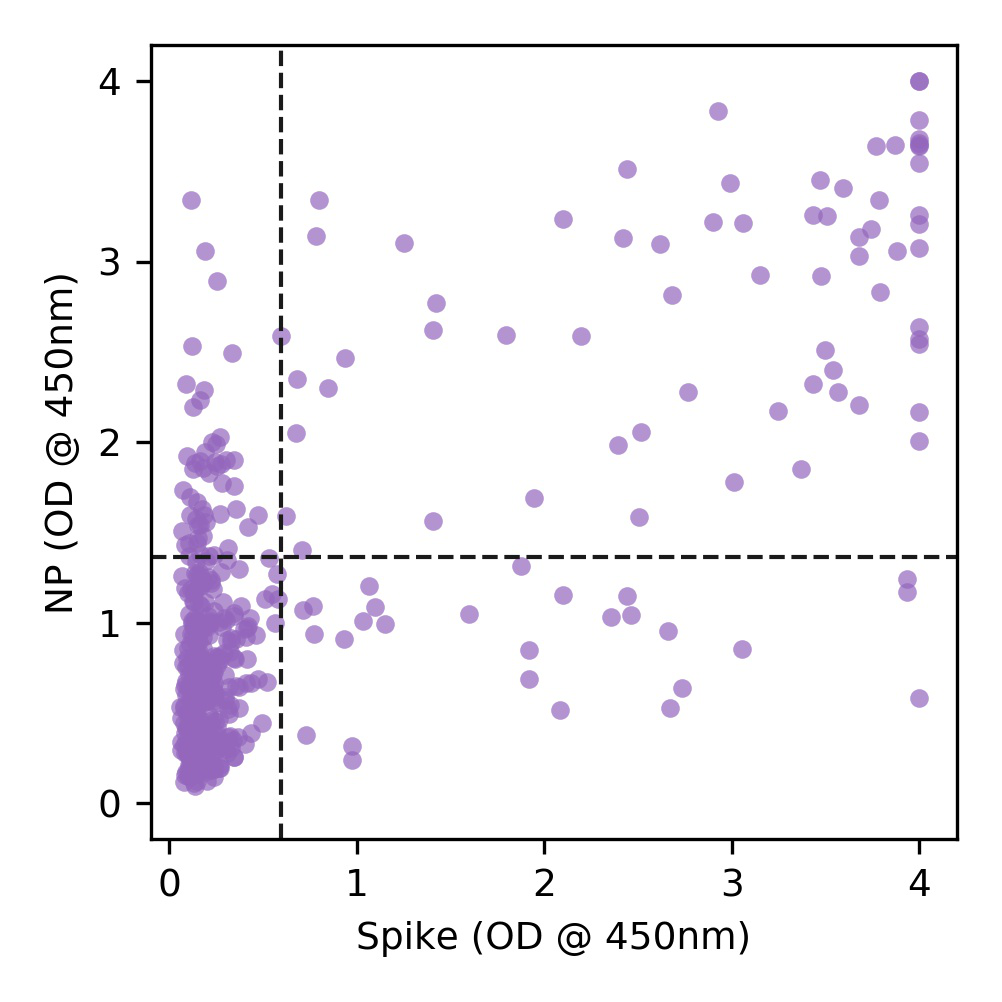

Supplement: FIG S1 [file msphere.00450-21-sf001.tif]
